# microRNA-1301-mediated inhibition of tumorigenesis

**DOI:** 10.3892/or.2011.1589

**Published:** 2011-12-12

**Authors:** LIN FANG, NING YANG, JIE MA, YONG FU, GUANG-SHUN YANG

**Affiliations:** 1 Fifth Department of Liver Surgery, Eastern Hepatobiliary Surgery Hospital, Second Military Medical University, Shanghai 200438; 2 Department of Laboratory Centre, Shanghai Tenth People’s Hospital, Tongji University School of Medicine, Shanghai 200072, P.R. China

**Keywords:** microRNA-1301, tumor, invasion, apoptosis, HepG2 cell

## Abstract

The relatively recent discovery of microRNAs has added a completely new dimension to the study of the regulation of tumor cells, but how they control cell behavior remains largely elusive. HepG2 cells were assigned to the miR-1301 group and the control group. RT-PCR, Western blotting, wound healing, the Transwell chamber migration and MTT assays, and apoptosis detection assays were used to analyze cell behavior of HepG2 cells after miR-1301 mimic transfection. Our study showed that miR-1301 was downregulated in HepG2 cells, and that miR-1301 inhibited migration and invasion of HepG2 cells and promoted cellular apoptosis after transfection with miR-1301 mimics. In addition, p53 mRNA and p53 protein expression was upregulated, and Bcl-2 and Bcl-xL mRNA and protein expression was downregulated in the miR-1301 group. These results indicate that miR-1301 may be an inhibitor of tumorigenesis in HepG2 cells.

## Introduction

microRNAs (miRNAs) are a class of approximately 20–25 nucleotide-non-coding RNAs that can regulate post-transcriptional gene expression. These miRNAs may degrade the target mRNAs or the translational repression of encoded proteins by partially binding to complementary target sites in messenger RNA (mRNA) 3′ untranslated regions (UTRs) (1).

Bioinformatic analyses have estimated that miRNAs may regulate as many as 30% of the human protein coding genes (2). In general, one gene can be repressed by multiple miRNAs, and one miRNA may repress multiple target genes. Using miRNA target prediction algorithms, hundreds of potential target mRNAs for a specific miRNA can be identified. miRNA target binding sites have been predicted to be present in almost any transcript. Many miRNAs are expressed in a tissue-specific manner and play pivotal roles in the control of proliferation and differentiation of different cell types (3–5).

Many miRNAs have been shown to be downregulated or upregulated in different tumors. miRNA expression profiles have been reported to undergo changes in papillary thyroid cancer (6), breast cancer (7), lung cancer (8), hepatocellular carcinoma (9), pancreatic adenocarcinoma (10) and colorectal cancer (11). In addition, deregulation of miRNAs may lead to oncogenesis and cancer progression. Studies have shown that miRNAs affect the expression of genes and pathways involved in cancer pathogenesis from initiation to metastatic disease (12,13). miRNAs are also involved in important homeostatic processes such as cellular proliferation and cell apoptosis (14,15).

In our previous study, we found that miR-1301 expression was lower in the HepG2 cell line than in the Qsg7701 cell line which is a normal liver cell line. In this study, we compared mRNA transcription, cell proliferation, migration ability, invasion ability and apoptosis rate in the HepG2 cell line before and after transfection with miR-1301 mimics to understand the role of miR-1301 in the control of HepG2 cell apoptosis.

## Materials and methods

### Cell line, culture and transfection

The human cell line HepG2 (Cell Bank of Chinese Academy of Sciences, Shanghai, China) were assigned to miR-1301 group and control group. The cells were maintained in monolayer cultures in high glucose Dulbecco’s modified Eagle’s medium (DMEM) containing 10% (v/v) fetal bovine serum (FBS) and 1% (v/v) penicillin at 37°C in a humidified atmosphere of 5% CO_2_ in air.

Cells at the log phase were harvested and a cell suspension of 3.0×10^4^ cells/ml was prepared in 96-well tissue culture plates. miR-1301 mimics (GenePharma Co., Ltd., Shanghai, China) were introduced into cells by the procedure according to the manufacturer’s instructions. miR-1301 mimics were mixed with Lipofectamine transfection reagent (GenePharma Co., Ltd.), incubated for 10 min, and diluted to a concentration of 50 nmol before transfection. The transfected cells were incubated for 24 and 48 h before harvest, and the samples were assayed. All transfections were carried out in triplicate. The primers for miR-1301 mimics were forward, UUGCAGCUGCCUGGGAGUGACUUC and reverse, AGUCACUCCCAGGCAGCUGCAAUU; negative control forward, UUCUCCGAACGUGUCACGUTT and reverse, ACGUGACACGUUCGGAGAATT.

### Reverse transcription-polymerase chain reaction (RT-PCR) analysis

Total RNA was extracted from human HepG2 cell lines using TRIzol reagent (Takara Biotechnology Co., Ltd.). Reverse transcription was performed using 1 μg of total RNA as a template and random hexamer as a primer. cDNA was amplified by PCR using specific paired primers. Expression of p65, β-catenin, p53, Tg737, Bcl-2, Bcl-xL, caspase-3, and caspase-8 in the cell line was detected by TaqMan stem-loop RT-PCR. TaqMan probes were used to quantify the levels of miRNA. All PCR reactions were run in triplicate.

### Western blotting

Cellular lysates were prepared as previously described (16). Total cell extracts were separated by SDS-polyacrylamide gel electrophoresis (SDS-PAGE) and transferred to nitrocellulose membranes. After saturation overnight in phosphate-buffered saline (PBS) containing 10% (w/v) skim milk with constant shaking, the nitrocellulose membrane was cut into strips and individually incubated with 1 ml of human serum diluted 1:500 in PBS/10% (w/v) skim milk at 37°C for 2 h. Each strip was washed three times with PBS/0.1% (v/v) Tween-20 and incubated with peroxidase-conjugated anti-human IgG (diluted 1:2000) for 2 h at room temperature. After washing, the reaction was developed with 0.5 mg/ml diaminobenzidine in PBS. The reaction was terminated with water. Positive and negative control sera were included in each experiment.

### Wound healing assay

For *in vitro* scratch assays, HepG2 cells were seeded and grown in 6-well plates at a density of 3×10^4^ cells/well in growth medium until they reached a confluence of ~80%. A scratch was made through each well using a sterile pipette tip, and cells were monitored under the microscope (magnification, ×150) for 0, 12, 24 and 48 h after wounding at 37°C in 5% CO_2_. Images of cells were captured at the same position before and after incubation to document the repair process. The experiments were repeated twice and representative pictures are shown.

### Transwell chamber migration assay

The Transwell migration assay was performed in a 24-well Transwell chamber system. The filter was washed with the same medium and placed between the lower and the upper chambers. HepG2 cells were trypsinized, resuspended in DMEM with 15% (v/v) FBS and transferred to the upper chambers. The chambers were incubated at 37°C in 5% CO_2_. After 48 h, the filter was removed. Cells were stained with eosin and then with thiazide dye. The upper surface of the filter containing non-migrating cells was cleared using a wet cotton swab. Five fields of each well were randomly gated and counted.

### MTT assay

The MTT assay was performed in HepG2 cells that were seeded and grown in 6-well plates at a density of 5×10^4^ cells/well. Cells were grown in growth medium until they reached a confluence of ~60%. miR-1301 mimics were diluted to concentrations of 10, 20, 40, 60, 80 and 100 nmol for transfection. The transfected cells were incubated in 96-well microculture plates at 37°C and maintained in humidified air with 5% CO_2_. After 24-, 48- and 72-h of transfection, MTT was added to all wells. The optical density (OD) of each well was measured with a microplate spectrophotometer at 490 nm. The inhibitor rate (IR) of HepG2 cell proliferation was calculated by the equation: IR = (1-OD_treated well_/mean OD_control well_) × 100%.

### Effect of miR-1301 transfection on HepG2 cell apoptosis

After removing the medium, HepG2 cells were washed with PBS. The reaction product was prepared according to the manufacturer’s instructions (Trevigen, Inc., Gaithersburg, MD, USA) and added. Cells were viewed under a fluorescence microscope through a dual pass filter allowing to visualize the Annexin-V-FITC positive and the propidium iodide positive cells in the same field according to the manufacturer’s instructions (Trevigen, Inc.).

### Statistical analysis

All data are presented as the mean ± SD. Statistical significance was calculated using the unpaired Student’s t-test. Significance was accepted at P≤0.05. All analyses were performed using SPSS version 13.0 (SPSS Inc., Chicago, IL, USA).

## Results

### miR-1301 regulates p53, Bcl-2 and Bcl-xL gene expression

**Figure 1:**
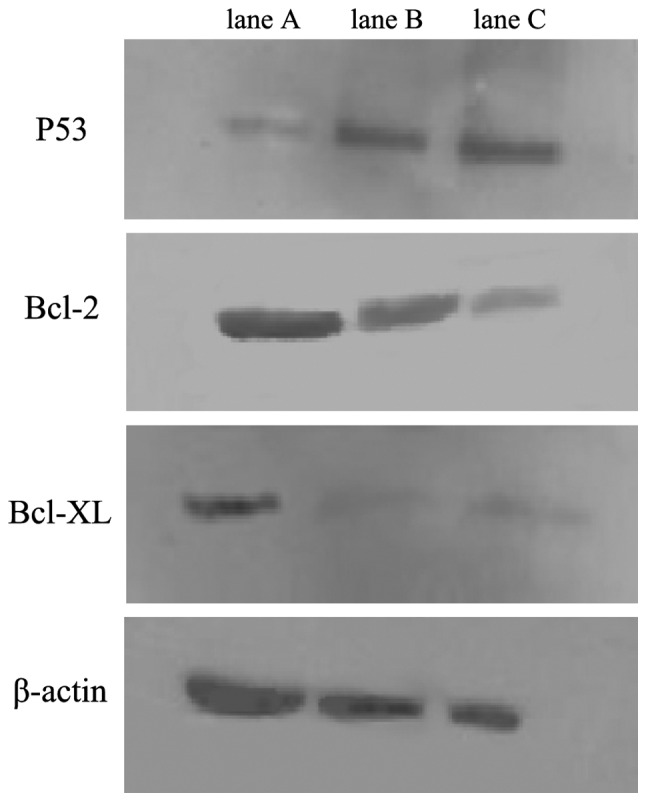
Protein expressions of p53, Bcl-2 and Bcl-xL after miR-1301 transfection. Western blotting shows that p53, Bcl-2 and Bcl-xL protein levels were altered. p53 protein expression increased in miR-1301 group, and Bcl-2 and Bcl-xL protein expression decreased in the miR-1301 group. Expression of β-actin was used as a control. Lane A, control group; lane B, miR-1301 group; lane C, Qsg7701 cells (normal liver cell line).

In this study, we found that p53 gene expression was upregulated in the miR-1301 group, and that Bcl-2 gene and Bcl-xL gene expression was downregulated when compared with the untransfected control group (Table I). Western blot analysis showed that levels of p53 protein increased, while levels of the Bcl-2 and Bcl-xL proteins decreased in the miR-1301 group (Fig. 1), indicating that miR-1301 may regulate HepG2 cell proliferation/apoptosis through apoptotic gene expression or tumor suppressor gene expression. There were no significant differences in p65, β-catenin, Tg737, caspase-3 and caspase-8 expression levels between the miR-1301 group and the control group.

### miR-1301 inhibits the migration and invasion ability of HepG2 cells

**Figure 2:**
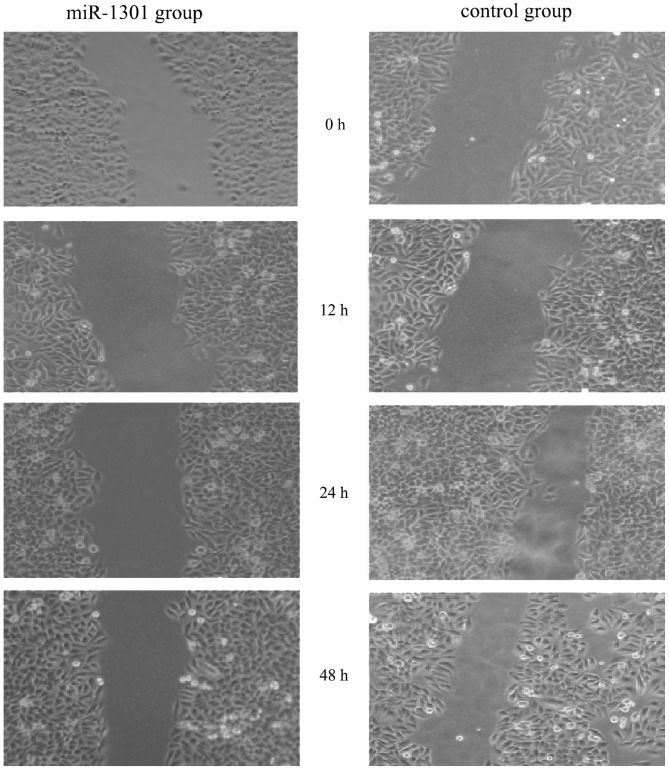
miR-1301 inhibits the migration of HepG2 cells in a scratch test. The scratch test was used to investigate the wound healing ability at 0, 12, 24 and 48 h. We observed that the proliferation and migration ability of HepG2 cells in the wounded area was reduced in the miR-1301 group after a 24 and 48 h repair period when compared with the control group. There was no difference between the miR-1301 transfected and the control group at 12 h.

**Figure 3:**
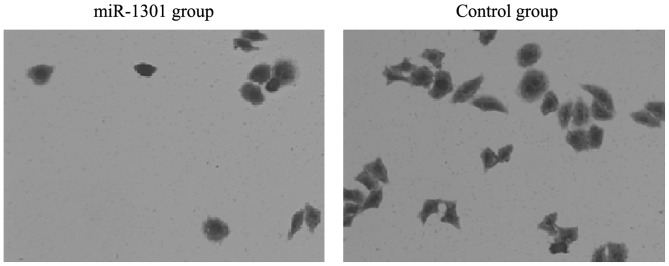
miR-1301 inhibits the invasion of HepG2 cells in the Transwell chamber assay. Cell invasion ability was analyzed by the Transwell chamber assay 48 h after miR-1301 transfection. The results showed that the number of migration cells in the miR-1301 group was less than that in control group. This indicated that the invasion ability of HepG2 cells might be inhibited by miR-1301.

**Figure 4:**
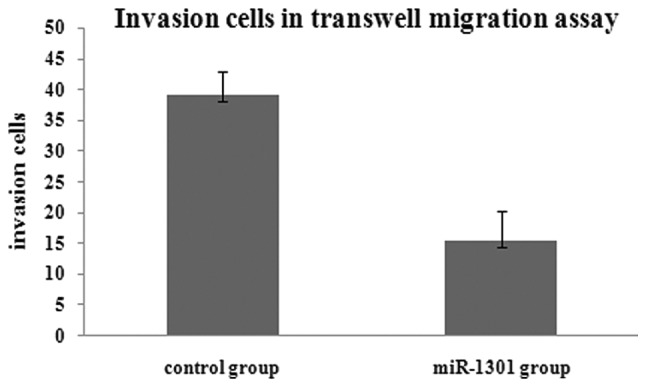
The number of migration cells in transwell chamber assay. The results showed that the number of migration cells in transwell chamber assay 48 h after miR-1301 transfection was less than that in control group (P<0.05). ^*^P<0.05 compared with the control group.

The scratch test is a useful method to investigate wound healing ability. Our results showed that the capacity for proliferation and migration of HepG2 cells into the wounded area was reduced in the miR-1301 group after a 24 and 48 h repair period (Fig. 2). There was no difference between the miR-1301 group and the control group at 12 h. The results of the transwell migration assay showed that the number of migration cells in the miR-1301 group was less than that in the control group. This indicated that the invasion ability of HepG2 cells might be inhibited by miR-1301 (Figs. 3 and 4).

### miR-1301 inhibits cell proliferation

**Figure 5:**
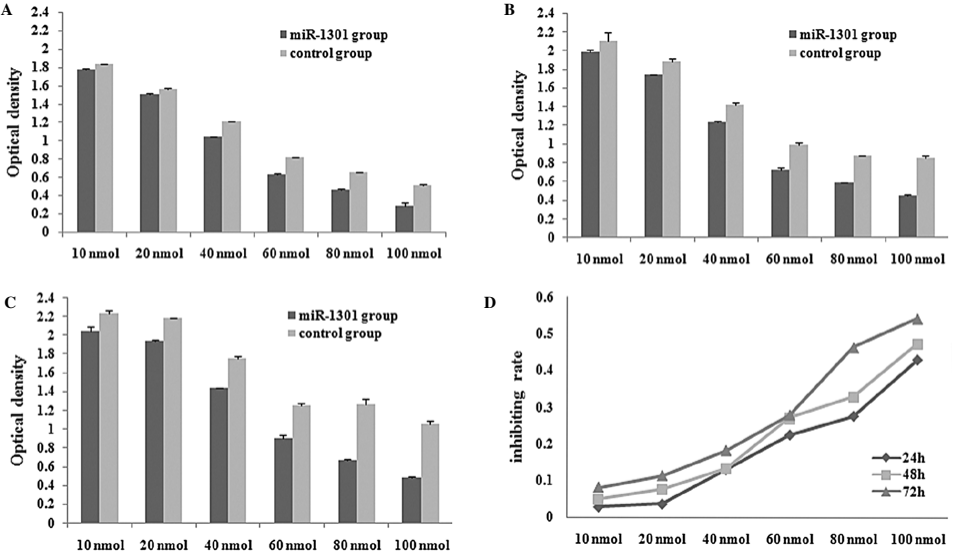
miR-1301 inhibits cell proliferation. The MTT assay was performed to monitor the proliferation rate of HepG2 cells after miR-1301 transfection. The optical density of each well was measured with a microplate spectrophotometer at 490 nm. The optical density of the miR-1301 group decreased at 24 h (A), 48 h (B), and 72 h (C) after transfection. The proliferation rate of cells decreased with increasing concentrations of inhibitor and increasing transfection time (D).

The MTT assay was performed to monitor the proliferation rate of HepG2 cells after transfection with varying concentrations of miR-1301 (10, 20, 40, 60, 80 and 100 nmol). The optical density of each well was measured with a microplate spectrophotometer at 490 nm at 24 h (Fig. 5A), 48 h (Fig. 5B), and 72 h (Fig. 5C) after transfection. Compared with the control group, the optical density of the miR-1301 group decreased at 24, 48 and 72 h. The proliferation rate of HepG2 cells was dependent on the concentration and time of transfection, with higher concentrations of miR-1301 and longer transfection times inhibiting cell proliferation (Fig. 5D).

### miR-1301 promotes cell apoptosis

**Figure 6:**
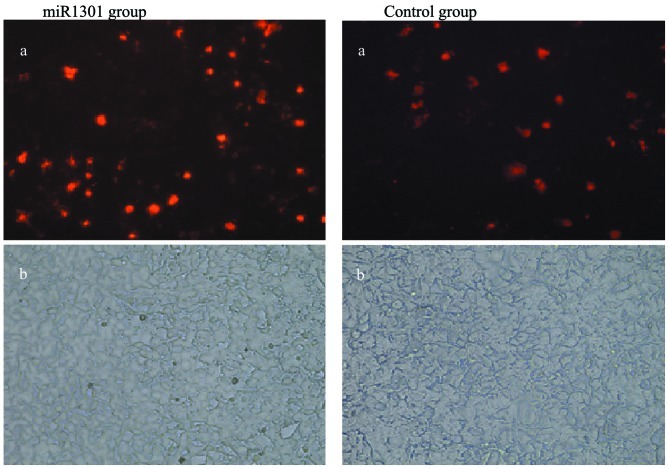
miR-1301 promotes cell apoptosis. Apoptosis of HepG2 cells was observed using fluorescence microscopy through a dual pass filter allowing to visualize the Annexin-V-FITC positive and the propidium iodide positive cells in the same field. The results showed that apoptosis of HepG2 cells increased 48 h after transfection of miR-1301 mimics in the miR-1301 group (Aa) when compared with the control group (Ba). (Aa) Apoptosis cells of the Annexin-V-FITC positive cells in the miR-1301 group; (Ab) cells excited by normal light in the miR-1301 group; (Ba) apoptosis cells of the Annexin-V-FITC positive cells in the control group; (Bb) cells excited by normal light in the control group.

HepG2 cell apoptosis was observed under a fluorescence microscopy. The results showed that HepG2 cell apoptosis increased in the miR-1301 group 48 h after transfection with miR-1301 mimics when compared with the control group (Fig. 6).

## Discussion

In this study, we found that miR-1301 expression was low in HepG2 cells. In addition, cells transfected with miR-1301 exhibited an upregulation of p53 gene expression, a downregulation of Bcl-2 and Bcl-xL gene expression, and increased apoptotic cell death. Moreover, miR-1301 reduced the cell migratory and invasive function of HepG2 cells. We therefore speculate that miR-1301 may be a tumor inhibitor.

The function of miRNAs has attracted much attention and research interest. Recent studies have shown that some miRNAs may behave as oncomiRs or tumor suppressors (17–19). These differing expressions of miRNAs could lead to different human cancers. miRNAs that are downregulated in cancers are usually termed tumor suppressors, while miRNAs that are upregulated in cancers are classified as oncogenes.

In this study, miR-1301 inhibits the proliferation of HepG2 cells. A number of miRNAs involved in regulating proliferation and growth have been identified through linkage with cancer phenotypes (20,21). Let-7 is known to control the timing of proliferation and differentiation in *C. elegans* (22). Let-7 has also been shown to repress Ras and c-Myc expression. Akao and colleagues (23) reported that Let-7 was greatly reduced in lung cancer and may facilitate high levels of Ras expression, and hence may act as a potent growth suppressor and tumor suppressor in normal cells. miR-372 and miR-373 have been shown to interfere with p53 function. They can also act as a promoter of tumors, and as oncogenes in testicular germ cell tumors (24). miR-125a has been shown to act as a regulator of the p53 gene, and may be add to the growing list of miRNA with oncogenic targets (25). The results of our study show that miR-1301 can inhibit the proliferation of HepG2 cells. The p53 gene was upregulated after transfection with miR-1301 mimics. We presume that miR-1301 may also interfere with p53 function, hence affecting cell proliferation. We also found that miR-1301 interfered with the migration and invasion ability of HepG2 cells, but the reason for this action remains unclear.

In this study, miR-1301 promoted apoptosis in HepG2 cells, and miRNAs have been shown to induce tumor apoptosis (26). Cimmino and colleagues (27) found that miR-15 and miR-16 could induce apoptosis by targeting Bcl-2 mRNA. Other studies (28,29) showed that there was a correlation between the expression of Bcl-2 and an absence of miR-15 and miR-16, which is important in the regulation of apoptosis in chronic lymphocytic leukemia. The Let-7 family of microRNAs inhibits Bcl-xL expression and potentially induces apoptosis in human hepatocellular carcinoma (30). Bcl-2 and Bcl-xL are anti-apoptotic proteins, which oppose the progression of apoptosis. These proteins inhibit apoptosis through the maintenance of mitochondrial membrane integrity and by binding to pro-apoptotic Bcl family members (31). Our study showed that miR-1301 promotes apoptosis, and that Bcl-2 and Bcl-xL genes were downregulated by miR-1301 at the same time. In our study, Bcl-2 and Bcl-xL gene expression were inhibited by miR-1301. We therefore conclude that miR-1301 may affect cell apoptosis through Bcl-2 and Bcl-xL.

Although we observed that miR-1301 could affect the proliferation and invasion ability and promote apoptosis in HepG2 cells, the true reason for this function remains unclear. First, it is unclear which genes are controlled by miR-1301 and how these genes are regulated. In addition, the mechanisms of cellular apoptosis and invasion remain unclear. Finally, our study was only performed in HepG2 cells, and we did not perform experiments to find target genes of miR-1301, which we are currently investigating.

In conclusion, our study indicates that miR-1301 reduces cellular proliferation, migration and invasion, and promotes cell apoptosis. We speculate that miR-1301 may be a tumor inhibitor. Further studies are needed to find target genes of miR-1301 and mechanisms of cellular apoptosis that are regulated by miR-1301, with the aim of preventing and treating tumors with related miRNAs.

## Figures and Tables

**Table I:** ΔCt values of expression genes between the miR-1301 and the control groups.

Group	miR-1301	p65	β-catenin	p53	Tg737	Bcl-2	Bcl-xL	Caspase-3	Caspase-8
miR-1301	−1.25±0.32^a^	5.26±0.26	4.60±0.30	5.05±0.14^a^	3.27±3.69	3.54±0.40^a^	−1.17±0.30^a^	3.35±0.52	4.26±0.46
Control	3.62±0.11	5.10±0.20	3.94±0.26	5.95±0.05	0.82±0.23	0.98±0.11	−3.70±0.23	2.75±0.25	4.68±0.02
t-value	−21.141	0.829	3.508	−16.043	1.945	11.227	20.221	2.651	−1.482

a P<0.05 vs. the control group.
